# Biopesticide Encapsulation Using Supercritical CO_2_: A Comprehensive Review and Potential Applications

**DOI:** 10.3390/molecules26134003

**Published:** 2021-06-30

**Authors:** Dário Rodrigues do Nascimento Junior, Antonio Tabernero, Elaine Christine de Magalhães Cabral Albuquerque, Silvio Alexandre Beisl Vieira de Melo

**Affiliations:** 1 Programa de Pós-Graduação em Engenharia Industrial (PEI), Escola Politécnica, Universidade Federal da Bahia (UFBA), R. Prof. Aristídes Novis, 02, Federação, Salvador 40210-630, Brazil; dario.rodrigues@ufba.br (D.R.d.N.J.); elainecmca@ufba.br (E.C.d.M.C.A.); 2 Departamento de Ingeniería Química, Universidad de Salamanca, Plaza los Caídos s/n, 37008 Salamanca, Spain; antaber@usal.es; 3 Programa de Pós-Graduação em Energia e Ambiente (PGENAM), Centro Interdisciplinar em Energia e Ambiente (CIENAM), Universidade Federal da Bahia (UFBA), Salvador 40170-115, Brazil

**Keywords:** encapsulation techniques, controlled release, supercritical fluid technology

## Abstract

As an alternative to synthetic pesticides, natural chemistries from living organisms, are not harmful to nontarget organisms and the environment, can be used as biopesticides, nontarget. However, to reduce the reactivity of active ingredients, avoid undesired reactions, protect from physical stress, and control or lower the release rate, encapsulation processes can be applied to biopesticides. In this review, the advantages and disadvantages of the most common encapsulation processes for biopesticides are discussed. The use of supercritical fluid technology (SFT), mainly carbon dioxide (CO_2_), to encapsulate biopesticides is highlighted, as they reduce the use of organic solvents, have simpler separation processes, and achieve high-purity particles. This review also presents challenges to be surpassed and the lack of application of SFT for biopesticides in the published literature is discussed to evaluate its potential and prospects.

## 1. Introduction

Over 10,000 years ago, in Mesopotamia, edible seeds were stored, marking the advent of agriculture as a way of providing food for the local population [1]. As the crops became bigger, agricultural pests, such as insects, fungi, bacteria, and weeds started to reduce the harvest yield [2]. To prevent those losses, some substances, known as pesticides, were used to control those organisms. Pesticides are any substance or mixture able to repel, eradicate, or mitigate any pest [3]. In the 1940s, during World War II, there was an expansion in synthetic pesticide usage, especially dichloro-diphenyl-trichloroethane (DDT), to limit the spread of malaria vectors, followed by carbamate and other organophosphate pesticides [4]. Since the intensive application of pesticides, agricultural productivity has increased exponentially. According to The Food and Agriculture Organization of the United Nations (FAO) data, approximately 4 million tonnes of pesticides were used globally or sold to the agricultural sector for crops and seeds in 2018. 

The negative effects on soil, air, water quality, agricultural products, and nontarget organisms became noticeable, alongside the evolution of pest resistance due to conventional synthetic pesticide use [2,3]. Less harmful alternatives have been used to control pests. As an example, any pest’s natural enemy can be used for biocontrol. Before this application, different species of organisms are tested against the target plant pathogen, and the most active species is considered for biocontrol. It also demands extensive knowledge of biocontrol mechanisms (e.g., competition, toxin production, and induction of resistance). Furthermore, properties such as biosafety, production requirements and conditions, and registration issues must be taken into account [5,6].

Another alternative to the use of synthetic pesticides is the application of biopesticides, which are based on naturally occurring living organisms, including animals, plants, and microorganisms [2]. The biopesticide can be retained by physical or chemical interactions within a matrix to provide chemical and physical protection against environmental factors and improve biopesticide stability by decreasing the volatility and reactivity of the active ingredient, a process named encapsulation [7,8]. There are several methods of encapsulation, such as emulsification, coacervation, and spray drying [7]. However, the use of supercritical fluid technology to encapsulate particles has also been explored due to its having several advantages, mainly low environmental impact and high purity [9,10]. Carbon dioxide is the most frequently used supercritical fluid since it has low toxicity, low cost, easy removal, and mild critical properties [11].

In this review, chemical and physical encapsulation methods are described, including their disadvantages and applications. Encapsulation methods using supercritical carbon dioxide are highlighted, with emphasis on the encapsulation of biopesticides.

## 2. Biopesticides

Biopesticides are types of natural pesticides consisting of natural products from living organisms, such as animals, plants, microorganisms, or genetically engineered organisms [2]. Biopesticides manage agricultural pests based on their biological effects on these organisms [12,13]. Some biopesticides can be based on substances that are toxic to the target pests, such as proteins or antibiotics. In fungal or bacterial sources, the mechanism of action can be also based on competition for space and nutrients on the surface of the host. Another main mode of action is by directly parasitizing the target pest [12,14]. In 2018, biopesticides comprised a small share of the total crop protection market globally, with a value of about USD 3 billion worldwide, accounting for just 5% of the total crop protection market [15].

Biopesticides can be classified into three main groups: (1) microbial pesticides, including bacteria, algae, fungi, viruses, or protozoa as active ingredients; (2) Plant-Incorporated-Protectants (PIPs), which are substances produced by plants due to changes in the genetic material; and (3) biochemical pesticides, referring to naturally occurring substances that control pests by non-toxic mechanisms [2,12,13]. The most commonly used microbial pesticide—almost 90% of the microbial biopesticides currently available [15]—is the insect pathogenic bacterium, *Bacillus thuringiensis* (Bt), which can lead to death by gut cell lysis in some insects. As for PIPs, for example, it is possible to insert a gene for a specific Bt pesticidal protein into plants’ genetic material, creating resistance against pest attack. This approach has been performed in rice, corn, tobacco, soybean, sugarcane, potato, alfalfa, tomato, brassica and cotton crops. Biochemical pesticides include pyrethrin, a secondary metabolite produced by plants, which can affect herbivore feeding, and two organic compounds supplied by neem (*Azadirachta indica*) trees, azadirachtin and salannin, that kill insects [12]. There are several patents concerning the use of natural products as biopesticides. Documented as US4668666, this patent relates to the use of nontoxic natural insecticides, focusing on improved pesticidal formulations using pyrethrum and/or synthetic pyrethroids [16]. The patent US9101143B2 provides an agricultural formulation comprising at least one volatile essential oil encapsulated in a non-volatile vehicle [17]. The patent US10058092 is related to botanical fumigant pesticide based on microencapsulated plant essential oils [18], and the patent US005413784A comprises the use of entomopathogenic fungus against insect pests [19]. Table 1 presents an overview of some of the commercial biopesticides available for sale.

Despite the low environmental impact of most biopesticides, since they present non-toxic or less toxic constituents, there are some concerns about the commercialization of natural pesticides, such as regulatory requirements, availability of the active ingredient, and stability of the formulation in storage, transport, and during application. To overcome the instability and lower number of active ingredients used, encapsulation technologies have been developed [32,33].

## 3. Encapsulation Technologies

Encapsulation is a physical process in which a core material or active substance, such as small solid particles, liquid droplets, or gases are entrapped in an encapsulating agent or wall material to completely or partially isolate the core material, improving its stability, by reducing its volatility and reactivity, and maintaining its viability against adverse environmental conditions, such as pH extremes [11,33,34].

When applied for biomedical purposes, drug encapsulation can reduce toxic side effects, provide drug protection against in vivo degradation and reduce the quantity of active ingredient used, due to its enhanced permeation and retention [34,35,36,37]. In the food industry, recent studies have been focused on encapsulating food-grade active ingredients, such as nutraceuticals, colorings, flavoring agents, enzymes, probiotics, and vitamins [38]. Encapsulation plays a major role in protecting these substances from hazardous external conditions, allowing their transport through the digestive system [39]. It also protects from oxidation reactions and the consequent undesirable taste [11,40]. For agricultural purposes, the process of pesticide encapsulation can reduce human exposure to active ingredients, reduce pesticide residues in agricultural products, increase protection from undesirable reactions and physical stresses, reduce the frequency of application, minimize environmental contamination and prolong the effective duration of non-persistent pesticides [3,7,8,41].

Depending on the materials and the technique used, different structures and morphologies are achieved in the encapsulation process. Solids, liquid droplets, and gas bubbles can be part of the shell material for liquid or gas core. The structure of the encapsulated particles can be spherical, in capsules, beads, monocore, multicore, multishell, or matrix [11,37].

To choose the proper encapsulation technique, the desired application, expected particle size distribution, biocompatibility of the particles, physicochemical properties of the active and the wall material, release mechanism, and process costs are evaluated [11]. There are many encapsulation techniques documented for biopesticides, classified by their chemical or physical processes. Among the chemical processes, the most explored are emulsion polymerization, miniemulsion, melt-dispersion, in situ polymerization, and coacervation. As for the physical processes, spray drying, fluidized bed coating, and ionic gelation encompass the most documented encapsulation techniques for biopesticides and natural substances that have the potential to be biopesticides [7,35,42,43,44,45] (Table 2).

In Table 2, a brief description of each encapsulation method is presented, and there is also information about the material used, particle size, and main disadvantages. Through emulsion polymerization, some natural products with the potential to be biopesticides were exemplified. Chang et al. (2013) [46] encapsulated carvacrol, present in the essential oils of oregano and thyme. In this study, the antimicrobial efficacy of the nanoemulsion was examined, but Campos et al. (2018) [50] explored the toxicity of carvacrol for undesirable invertebrates and nematodes that affect crops. By encapsulating, the low solubility in water of carvacrol was overcome. As the oil concentration increases in the formulation, the storage stability also increases, while the antimicrobial efficacy decreases. This problem can be surpassed by diluting the nanoemulsions before storage. This process was proven to demand low energy and non-complex processes, such as simple stirring. On the other hand, a considerable amount of surfactant is required. Feng et al. (2020) [51] used emulsion polymerization to encapsulate D-limonene, an active ingredient from essential oils of citrus fruits. This substance has documented fungicidal and insecticidal activity, by possibly damaging the cytoplasmic membrane of organisms. D-limonene undergoes oxidative degradation and volatilization so, by encapsulating, it can increase its chemical stability and allow a slow release of this active compound. In this paper, the nanoformulation showed great control of four agricultural pathogens and the fungicidal activity was higher after loading. Though a similar encapsulation technique, demanding more energy, in a process called miniemulsion polymerization, Wahba (2020) [52] encapsulated tea tree oil, grape seed oil, and pomegranate fruit peel oil to evaluate their antifeedant activity against rice weevil (*Sitophilus oryzae*), which affect stored grains. This method was used to increase the water solubility, permeability and thermal stability of the essential oils without using organic solvents. Since a high-energy emulsification was performed by high shear stirring and ultrasonication, the formulation presented low polydispersity and the droplets did not coalesce. The mortaility of rice weevil was higher after loading.

Garlic essential oil has strong insecticidal activity; however, it has high volatility, strong odor, and low solubility in water. To overcome these drawbacks and increase the insecticidal effectiveness and shelf life, Yang et al. (2009) [54] loaded polyethylene glycol (PEG) with garlic essential oil by melt-dispersion. The formulation was tested against *Tribolium castaneum* and groups treated with free essential oil presented a rapid decrease in mortality over time, while the nanoparticles presented a gradual decrease in mortality over time. The insecticidal effectiveness was not affected by the 65 °C temperature held in the melt-dispersion process. Though in situ polymerization, Bagle et al. (2013) [56] loaded phenol aldehyde microparticles with neem oil, one of the most effective biopesticides. Neem oil contains azadirachtin, responsible for affecting the behavior and physiology of insects by blocking real hormones from working properly. The encapsulation process was performed to prevent the rapid degradation of neem oil, improve its stability in the environment and allow its slow release. The resulting particles presented spherical shape, with smooth morphology, no agglomeration, and thermal stability. 

By complex coacervation, Qiu et al. (2019) [61] entrapped *Metarhizium anisopliae*, a fungus with insecticidal effects against red imported fire ants (*Solenopsis invicta*), using gelatin and gum Arabic as wall materials. The microencapsulated conidia presented more resistance to UV exposure and longer lifespan. This can be explained by the mild temperature conditions under which complex coacervation occurs, unlike spray drying and melt-dispersion, preserving the conidia from thermal degradation. Additionally, the layer of gelatin and gum Arabic allows isolation from UV radiation and oxygen. Another fungus, *Trichoderma harzianium*, used to attack pathogenic plant fungi, was encapsulated. Muñoz-Celaya et al. (2012) [66] loaded maltodextrin and gum Arabic with *Trichoderma harzianium* conidia. Spray drying was chosen as the method to encapsulate and the use of maltodextrin and gum Arabic was essential to prevent thermal stress during the spray drying operation. This study stated that using the 1:1 blend of maltodextrin–gum Arabic, the best conidia survival rate was achieved (86%). The storage temperature is also important for spore survival. The lowest storage temperature yielded 8 weeks of storage. Pérez-Landa et al. (2020) [64] entrapped the photosensitive biopesticide Spinosad in chitosan/sodium lignosulfonate particles using the spray drying technique. Spinosad acts by disrupting nicotinic acetylcholine receptors of insects but has limitations when used in agriculture since it is photodegradable by sunlight or UV light. In this work, the photostability of Spinosad was enhanced with biopolymers that absorb UV rays. The in vitro results indicated that Spinosad release follows the zero-order kinetic model, with an initial burst effect and then slow release of the active ingredient.

Stephan et al. (2020) [69] investigated fluid-bed coating with liquid fermented biomass containing spores of fungi *Metarhizium brunneum, Cordyceps fumosorosea,* and *Beauveria bassiana* approved as an active substance for integrated pest management in Europe. Solid-state fermenters, which are the base for the production of most granules, have high contamination risks and complications for scaleup, so this work focuses on liquid fermented biomass with autoclaved millet as core material and a thin layer of fungal biomass as coating material. The efficacy of these granules was not tested in the field.

Entomopathogenic nematodes (EPN) were also encapsulated. Jaffuel et al. (2020) [72] produced alginate beads containing *Heterorhabditis bacteriophora* to protect maize plants from *Diabrotica balteata* larvae. Ionic gelation was used to encapsulate those EPN aiming the increase shelf life and survival after application. Through this technique, an EPN-alginate-glycerol solution interacted with a Ca^2+^-glycerol solution. This study tested EPN efficacy to control *Diabrotica* spp. but it was only conclusive in laboratory tests. Field applications depend on several environmental and application conditions. In the laboratory, the encapsulated EPN presented prolonged shelf life and controlled release, but the larval mortality remained the same as EPN in an aqueous solution.

Among the many encapsulation techniques, there are also those based on supercritical fluid technology (SFT). SFT has many advantages, such as reduced environmental impact, low waste toxicity, and enhanced product quality and safety [9]. This technique stands out in encapsulating thermolabile particles (i.e., those that can be readily destroyed or deactivated by heat) and for creating size-controlled particles [11]. 

## 4. Biopesticide Encapsulation Based On Supercritical Fluid Technology

Above the critical temperature and critical pressure of a fluid, it is named a supercritical fluid, a phase in which there is no transition between vapor and liquid states. The lack of transition between those two phases provides a great solubilizing capability to the supercritical fluid (SCF), which increases with higher pressure values at a constant temperature. SCF refers to a single-phase matter in a non-condensing state near the critical point, which is sensitive to changes in pressure and temperature, once the slightest variation in these parameters leads to noticeable changes in density, resulting in a more gaslike or liquidlike fluid [9,42,74,75].

Compared to other conventional encapsulation processes, the usage of SCF has advantages, such as easy separation of solute/solvent and tunable density. Furthermore, it reduces the use of toxic organic solvents and there is no need to carry out separation steps since this can be performed by depressurization [76,77]. 

SCF can be used as a solute, solvent or antisolvent, to extract substances from natural products, sterilize products using high-pressure treatment, micronize drugs and encapsulate active substances in a polymeric matrix [74]. The most widely used supercritical fluid in encapsulation processes is supercritical carbon dioxide (scCO_2_), due to its low toxicity, low cost, non-flammability, easy removal [11], permeability, and mild critical properties (critical temperature, T_c_ = 304.25 K and critical pressure, P_c_ = 7.38 MPa), so it is suitable for processing thermolabile compounds. On the other hand, scCO_2_ has some drawbacks, such as non-polarity and solute–solvent interactions, which can be formed due to carbon dioxide’s electron donor and acceptor sites [9,78,79].

Some supercritical fluid techniques have been considered to create polymeric micro or nanoparticles of biopesticides with narrow size distribution and high stability, such as Rapid Expansion from Supercritical Solution (RESS), Particle from Gas Saturated Solutions (PGSS), Supercritical Solvent Impregnation (SSI) Supercritical Assisted Atomization (SAA) and Supercritical Phase Inversion [74,77,80,81,82]. Table 3 illustrates some applications of supercritical technologies applied to biopesticides or natural products that could be potential biopesticides.

### 4.1. Rapid Expansion from Supercritical Solution (RESS)

**Figure 1:**
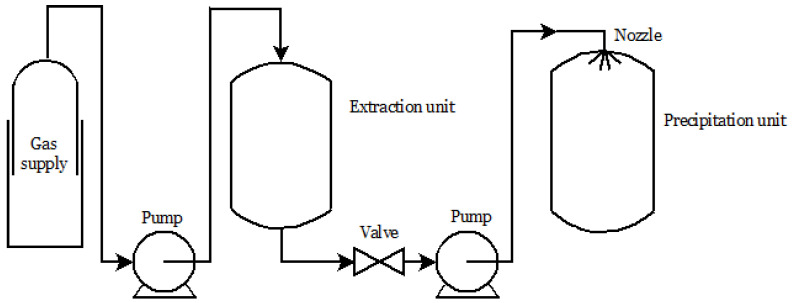
Experimental scheme for RESS.

Through Rapid Expansion from Supercritical Solution (RESS), fine particles can be produced by saturating the SCF with a solid in an extraction unit. Then, the solution is abruptly depressurized in a low-pressure chamber, leading to rapid nucleation of the substrate in fine particles, collected from the gaseous stream [74,93] (Figure 1). The morphology of the resulting solid material depends on the temperature, pressure drop, nozzle geometry, and other parameters, as well as on the chemical structure of the active ingredient [93]. 

Although RESS has been used to create films, nano, and microparticles, it is difficult to control the particle size of precipitates, due to the particle coalescence in the supersonic free jet generated in the precipitation vessel. To avoid the formation of liquid droplets, it is necessary to have previous knowledge of the variation of solid/polymer melting temperature with the pressure. Therefore, it is possible to determine the solubility of the particles in the SCF [78], since this process is suitable only for particles that are soluble in the supercritical fluid, excluding solid active compounds with high molecular weight and polar bonds, since they have low solubility in scCO_2_ [93,94].

This method has been explored for a wide range of materials, including polymers, dyes, medicines, and inorganic substances [74]. Using CO_2_ as a solvent, ethanol as a cosolvent, and polyethyleneglycol (PEG) as encapsulant material, Santos et al. (2013) [80] studied the encapsulation of anthocyanins extracted from jaboticaba skins. The encapsulation was carried out by the RESS process, and the optimal condition was at a temperature of 40 °C and pressure of 20 MPa.

Using the RESS process it is possible to encapsulate in liposomes an essential oil extracted from the rhizome of *Atractylodes macrocephala,* a traditional Chinese medicine (Table 3) [83]. This essential oil was dissolved in a mixture containing scCO_2_ and ethanol. As a result, particles were obtained with an average diameter of 173 nm and 82.18% of entrapment efficiency. Even though it was not the application in the previous work by Wen et al. (2010) [83], in Chu et al.’s (2011) [84] paper, some compounds of this oil presented pesticide effects against the common vinegar fly, *Drosophila melanogaster* L., suggesting that the potential of this oil as biopesticide can be assessed in further studies. 

### 4.2. Particle from Gas Saturated Solutions (PGSS)

**Figure 2:**
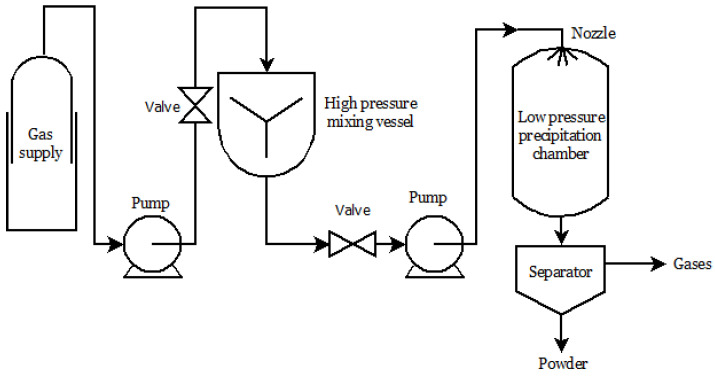
PGSS process scheme.

Particle from Gas Saturated Solutions (PGSS) is a process in which an SCF, usually scCO_2_, is dissolved in different materials of low melting temperatures. This technique has been used for some liquids and solids, such as powder coatings, monoglycerides, vitamins, antioxidants, fats, and food-related products [85]. The process is schematized in Figure 2. The solution or melt to be crystallized is mixed and saturated with the SCF at an appropriate pressure and temperature, and the solution is sprayed into a low-pressure precipitation chamber. After the depressurization, the dissolved carbon dioxide is released and expands; consequently, there is a cooling effect that promotes the formation of the microparticles [42,95].

Varona et al. (2009) [86] encapsulated lavender essential oil in PEG and octenyl succinic anhydride (OSA) starch, a chemically modified starch. Before processing in the PGSS plant, emulsion water in oil was prepared. PEG microcapsules achieved high encapsulation efficiency, ranging from 14 to 66%. Particles were spherical and a narrow particle size distribution was present of 21 to 49 µm. No data on the active ingredient release profile was obtained.

### 4.3. Supercritical Solvent Impregnation (SSI)

Using Supercritical Solvent Impregnation (SSI), it is possible to impregnate polymers with active compounds dissolved in supercritical fluids, especially scCO_2_. It is also possible to dissolve other substances to enhance the solubility of the active compound, acting as a cosolvent, or add a surfactant to improve its dispersion in the polymer [42].

**Figure 3:**
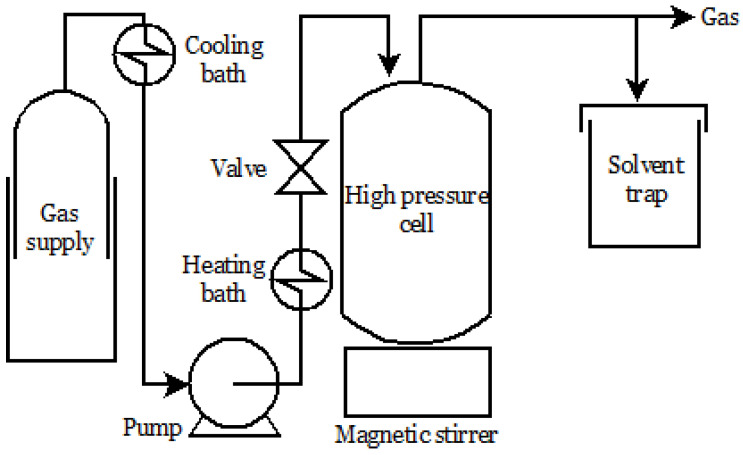
Supercritical impregnation apparatus.

Divided into three steps, the SSI process starts with the dissolution of active substances in SCF. After that, the supercritical fluid containing the active ingredient swells into a polymeric matrix, promoting the internal diffusion of the active compound followed by decompression, lowering the SCF density and allowing solute precipitation [96] (Figure 3).

Goñi et al. (2017) [88] incorporated two terpenic ketones, thymoquinone and R-(+)-pulegone, in low-density polyethylene (LDPE) by the impregnation of a supercritical solvent with CO_2_. The impregnated films presented a ketone concentration ranging from 2.25 to 5.59% (*w*/*w*) and a high insecticidal effect was obtained, with nearly 85% mortality against the weevil *Sitophilus zeamais*, which fell to 20–30% over the next seven days.

### 4.4. Supercritical Assisted Atomization (SAA)

**Figure 4:**
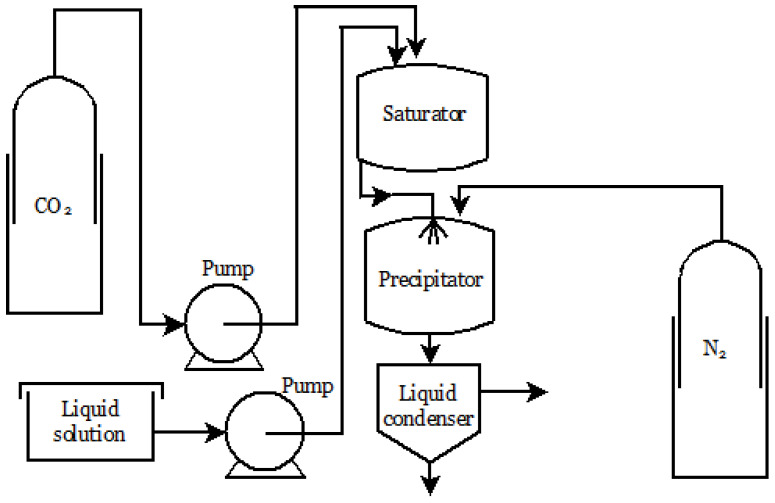
SAA apparatus.

In the Supercritical Assisted Atomization (SAA) process, scCO_2_ acts as a cosolute and is dissolved in a solution containing the active compound, forming an expanded liquid solution with reduced viscosity and surface tension, reducing cohesive forces [90]. This is a two-step atomization: first, there is pneumatic atomization, in which a pressure drop occurs at the nozzle outlet, and then there is decompressive atomization, by steadily delivering CO_2_ in the primary droplets. The microparticles are formed through the evaporation of the solvent and supersaturation of the solute in the droplets (Figure 4) [97].

This technique has been used with different compounds: active molecules, proteins, and polymers [81]. SAA can be applied with an organic and aqueous solvent, and it is a flexible and easily scalable process [97]. Even though this process presents some limitations for thermolabile compounds due to the high temperature required, this problem can be overcome by applying a vacuum in the precipitator [78]. Santo et al. (2014) [98] prepared liposomes from droplets formed by the atomization of an expanded liquid mixture of phospholipids, ethanol, and carbon dioxide. These droplets were coated by a lipid layer, resulting in a water-in-CO_2_ emulsion, which was transformed into liposomes by precipitation in the water pool at the bottom of the vessel. The mixer and vessel pressure varied from 12.5 to 17.5 MPa, and the temperature was set at 70 °C. It was possible to form particles with sizes ranging between 130 ± 62 and 294 ± 144 nm and encapsulation efficiencies from 85 to 90% were obtained. Adami et al. (2011) [99] produced microparticles of two thermolabile compounds: bovine serum albumin (BSA) and poly L-lactide (PLLA). Spherical particles were obtained with sizes ranging from 1 to 1.5 µm. The precipitation pressure was 0.05 to 0.065 MPa to avoid coalescence and the saturation pressure ranged from 8 to 10.5 MPa. The precipitation occurred from 30 to 80 °C, and the saturator temperature fluctuated between 60 and 81 °C. Using polyethylene glycol (PEG) and polyvinylpyrrolidone (PVP) as biopolymers, rotenone was encapsulated by the SAA process in Martin et al.’s (2013) work [90]. Rotenone is an organic molecule that occurs naturally from the seeds and stems of several plants, such as *Derri elliptica* and *Tephrosia vogelii*. The spherical particles obtained had a mean diameter varying from 0.6 to 1.5 µm with an encapsulation efficiency near 100%.

### 4.5. Supercritical Antisolvent Fractionation (SAF)

Through Supercritical Antisolvent Fractionation (SAF), the SCF, mainly scCO_2_, is used as an antisolvent: it dissolves the organic solvent and eliminates undesired compounds. As a result, there is an enrichment of the active ingredient [91].

**Figure 5:**
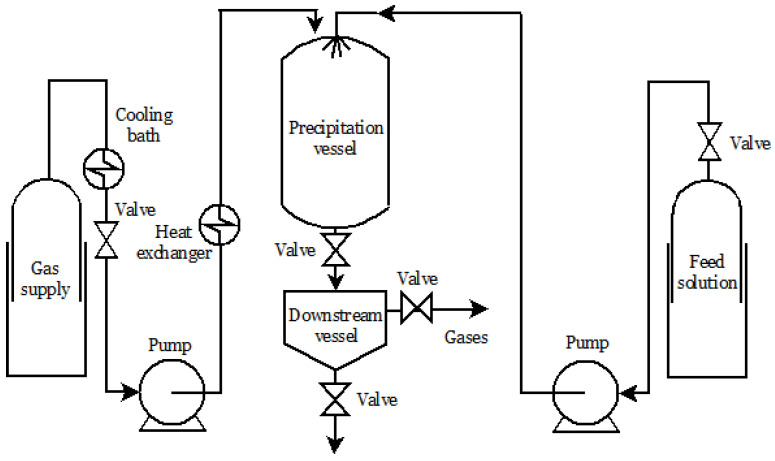
Scheme of a SAF plant.

Since scCO_2_ has a non-polar nature, it is possible to fractionate polar compounds of interest in an organic solution containing various components. This happens during the contact between the SCF and the liquid mixture in a pressurized vessel. The liquid solution, dispersed via spray, can be fractionated and precipitated in a high-pressure vessel. CO_2_ is recovered by decompression [76,91] (Figure 5).

Martin et al. (2011) [91] extracted and concentrated ryanodol, an insecticidal compound present in the plant *Persea indica*, using the SAF method. Before the concentration by SAF, the extraction occurred by using ethanol. This operation was performed at 15.0 MPa and 35 °C. Ryanodol concentration went from 7.5% (*w*/*w*) in the dry extract to 37.7% (*w*/*w*), after SAF technique.

### 4.6. Supercritical Phase Inversion and Supercritical Drying

Supercritical Phase Inversion offers the possibility to create porous membranes of polymers that can be loaded with active ingredients. In this technique, a liquid–liquid demixing phenomenon is produced from a polymeric solution by using scCO_2_ as a non-solvent to steadily remove the organic solvent. The use of scCO_2_ in this phase inversion avoids the collapse of the pore structure due to its low surface tension and gaslike diffusivity. Moreover, a subsequent membrane drying process is not required. The membrane morphology can be controlled by changing the operating conditions [100] and compounds can be included in the membrane. This process starts with producing polymer solutions by solubilizing the polymer in an organic solvent that should have a proper affinity with scCO_2_. The final solution is placed in stainless steel caps and processed in a vessel containing scCO_2_, filled from the bottom up to the desired pressure. Then, this vessel is depressurized, forming dried membranes [101].

Baldino et al. (2021) [92] used quercetin, a plant flavonoid, as a fungicide against *Kluyveromyces lactis* and *Yarrowia lipolitica*. Membranes of cellulose acetate were produced by supercritical phase inversion to load quercetin with different polymer concentrations (5%, 10%, and 15% *w*/*w*), pressures (10 and 20 MPa), and temperatures (45 and 50 °C). Aerogels are ultralight materials with high porosity. This type of material is obtained by drying a gel. The gel can be produced by different gelation methodologies in bead- or monolithlike form, and from different inorganic or organic compounds. After that, the gel must be dried with any technique. In this context, the use of scCO_2_ helps to preserve the gel’s internal network due to its low surface tension. The affinity of the solvent and scCO_2_ must be high. Then, if a hydrogel has to be dried (water-scCO_2_ affinity is low), a multistep solvent exchange must be performed with a different organic solvent before the drying process to obtain an alcogel [102,103,104].

Aerogels can be loaded with any compound in different steps. The molecule can be included in the precursor solution, during the gel formation, in the alcogel, during the supercritical drying, or even after obtaining the aerogel by any impregnation methodology, such as supercritical impregnation. These phenomena have been reviewed in [103,105,106]. Registered by the number US7674476B1, this patent is related to the use of aerogels as a carrier for active ingredients in agriculture and/or veterinary medicine. The active materials can be insecticides, herbicides, fungicides, acaricides, rodenticides, piscicides, molluscicides, nematicides, bactericides, and/or parasiticides [107].

### 4.7. Supercritical Anti-Solvent Precipitation (SAS)

A potential SFT for biopesticide encapsulation is Supercritical Anti-Solvent Precipitation (SAS), in which scCO_2_ is used as antisolvent, so it has to be miscible with the chosen liquid solvent. This method aims to create a free-flowing powder, and a polymer can be added to the solution to encapsulate the particles [76]. SAS treated material can range from nanoparticles to microparticles and it can be amorphous or semi-crystalline [108].

In this process, CO_2_ is pumped at a constant rate to the precipitation chamber until it reaches the desired pressure (Figure 5). Then, the temperature is set and the pure solvent is sent to the precipitator through a nozzle. After that, the liquid solution containing the active ingredient dissolved in the selected solvent is injected. As a result of the supersaturation, solute precipitation occurs on a filter, while the solvent/antisolvent mixture is recovered. The use of scCO_2_ eliminates the solvent residues. Finally, after the washing step, there is a depressurization in the precipitator to atmospheric pressure to collect the resulting powder [82,109,110].

Compared to other techniques such as RESS and spray-drying, the particles formed by SAS present a narrower particle size distribution and higher specific surface area [110]. Among the limitations of SAS, some stand out, such as the difficulty in working with molecules soluble in CO_2_ or the solvent/antisolvent mixture, and with hydrophilic compounds, since there is a notable difference between water and CO_2_ solubility in SAS conditions [82].

Oliveira et al. (2017) [111] encapsulated passion fruit seed oil in poly(lactic-co-glycolic) acid (PLGA) by the SAS method to maintain the antioxidant and antimicrobial activities. The organic phase, containing oil, PLGA, and dichloromethane, and CO_2_ were fed into the precipitation chamber, where fast solubilization of the organic solvent in CO_2_ occurred, precipitating the oil/PLGA mixture. After depressurization, the particles collected presented a size distribution from 721 to 1498 nm, with the entrapment efficiency varying from 67.8 to 91%. Differential Scanning Calorimetry (DSC) tests showed that lower temperatures (35 °C) prevented thermal degradation of the oil.

Although there is a lack of biopesticide encapsulation using supercritical technology in the literature, SFT could be applied to compounds that were encapsulated by other methods to explore their advantages. As an example, it is possible to evaluate the possibility of creating an inclusion complex of pesticides with cyclodextrins by the SAS technique, since there is a previous study [112] using cyclodextrins to extract pesticides from the soil.

### 4.8. Challenges of Supercritical Fluid Technologies

One of the main challenges of using SFT is the solubility or insolubility between the active substance and the carrier material in the supercritical fluid, especially CO_2_ because it indicates the amount of active compound that can be entrapped in the process. Once the solubility data is well-known, the shelf life can be assessed, since it is possible to understand the susceptibility of the active compound to degradation processes by segregation out of the carrier [45].

The phase equilibria modeling under pressure for SCF has not presented good predictability, especially when multiple phases, such as cosolvents and polymeric carriers, are evolved in the operation. Therefore, it is necessary to have extensive experimental data available to scale up and allow these techniques to be reproducible [77].

Although supercritical fluid techniques for particle formation have been successfully used in research for many years, there are still many issues concerning their scaleup. In atomization processes, the length-to-diameter ratio and fluid velocity are very important to determine the final particle size. In this context, different approaches can be followed. The first one is the use of several plants with the same dimensions (that is, numberingup), but that approach involves different drawbacks, such as keeping the same pressure drop in every nozzle or avoiding nozzle blockage due to the Joule–Thomson effect in every piece of equipment. Therefore, it seems more appropriate to use only one device, keeping constant the governing process dimensionless numbers, such as Reynolds, Weber, or Ohnesorge, and as a consequence, the different governing forces involved will be controlled [113,114].

The following issue concerns particle recovery. In this case, filtration is the more adequate technique, but with some problems, such as harvesting from the filters or even the residence time. This drawback can be resolved by using more collection filters, as was proposed by Clavier and Perrut [115]. Finally, it is important to design a recycling system to recover the used supercritical fluid, and a process separation step should always be included to remove cosolvents, antisolvents, or in this topic, pesticide traces. This issue is related to every supercritical fluid technique (drying with supercritical fluids or for particle formation).

Perhaps the most important thing is to perform the scaleup, fulfilling the Good Manufacturing Practices (GMPs). Supercritical fluid techniques are high-pressure processes with a high risk. Therefore, automatization systems, the use of “clean” rooms for the vessels, and fluid management should be taken into account. Moreover, special care has to be taken concerning blockages of pipes or nozzles due to the Joule–Thomson effect, as well as with leaks.

Although it is difficult to fulfill all the previously explained issues, it should be taken into account that extraction processes with scCO_2_ are already used on an industrial scale and that some APIs (Formulcoat, Formulplex, and Formuldisp) are already under pre-formulation [116].

## 5. Conclusions

Biopesticides have gained recognition in the marketplace, since they present several advantages, such as less toxicity to crops and nontarget organisms compared to extensively used synthetic pesticides. However, regulatory requirements, the stability of the formulation, availability, and standardization of the active compound limit their commercial success, which is reflected in the tiny selection of products presented in this review.

Regarding the physical and chemical stability of the active ingredients of biopesticides, this work summarized the most common encapsulation techniques, highlighting the materials used, particle size, and their effect on the stability of the active compound. Supercritical fluid technology, especially that using supercritical carbon dioxide, stands out among other encapsulation technologies, due to the reduction in toxic organic solvents and ease of separation by depressurization.

Challenges regarding the use of supercritical fluid technologies were presented, such as solubility in the SCF, scaleup, multiphase equilibria, and product recovery concerns. Furthermore, studies must be carried out to investigate if encapsulation processes can damage biopesticide activity, especially under supercritical conditions.

## Figures and Tables

**Table 1:** Commercial biopesticides available for sale.

	Active Ingredient	Target	Application	Product	Manufacturer	Reference
Microbial Pesticides	ABTS-1857 strain of *Bacillus thuringiensis aizawai* (Bta)	Caterpillar pests on vegetables, fruits, nuts, row crops and turf.	Dry flowable and water-dispersible granule formulations	XenTari^®^	Valent BioSciences	[20]
	ABTS-351 strain of *Bacillus thuringiensis kurstaki* (Btk)	Caterpillar pests on vegetables, fruits, nuts, cotton, oil, palm and corn.	Water dispersible granule to be dispersed after mixing with water	DuPel^®^	Valent BioSciences	[21]
	*Bacillus thuringiensis* BT	Pests of Orthoptera, Coleoptera, Diptera, Hymenoptera, and especially Lepidoptera.	Water-dispersible powder	Bactospeine	Xi’an NEO Biotech	[22]
	*Bacillus popilliae*	Japanese beetle grubs.	Water-dispersible powder	Milky Spore Powder	St. Gabriel Organics	[23]
	*Chromobacterium subtsugae*	Armyworms, aphids, Asian citrus psyllid, mites, spotted wing drosophila, thrips and whiteflies.	Water-dispersible powder	Grandevo^®^	Marrone Bio Innovations	[24]
	*Bacillus subtilis*	White mold and citrus canker.	Aqueous solution	Serenade^®^	Bayer	[25]
	WYEC 108 strain of *Streptomyces lydicus*	Soil-borne and foliar diseases across multipxle crops.	Water-dispersible powder	Actinovate^®^	Valent	[26]
Biochemical Pesticides	Neem oil extracted from *Azadirachta indica*	A wide variety of insects, such as beet armyworm, aphids, thrips, whiteflies, mites, fungus gnats, beetles, moth larvae and nematodes.	Concentrated aqueous solution	Neem Oil	Bonide	[27]
	Tea tree oil	Powdery mildew on capsicums, cucurbits, grapes and tomatoes	Emulsifiable Concentrate	Timorex^®^ Gold	Stockon	[28]
	Canola oil	A wide range of insects and eggs.	Emulsifiable Concentrate	Take Down Garden Spray	Monterey	[29]
	Potassium bicarbonate	Larvae of over 40 crops	Water-soluble powder	Kaligreen^®^	Brandt	[30]
	Extract from *Reynoutria sachalinensis*	Used for disease control, such as black spot, gray mold, crown rot and powdery mildew	Emulsifiable Concentrate	Regalia^®^	Marrone Bio Innovations	[31]

**Table 2:** Encapsulation techniques for biopesticides.

	Method	Description	Drawbacks	Particle Size	Materials	Active Component	Reference
Chemical Processes	Emulsion Polymerization	An organic phase is emulsified in an aqueous phase. Polymerization starts with a water-soluble initiator. Fine oil droplets are spontaneously formed when the surfactant moves from the organic phase to the water phase, resulting in oil-in-water (*o*/*w*) emulsion [46,47,48]. When an aqueous phase is emulsified in the organic phase of very low polarity, it results in a water-in-oil (*w*/*o*) emulsion. This process is referred to as inverse emulsion polymerization [47]. To achieve greater colloidal stability, emulsifiers are added at concentrations equal to or above their critical micelle concentration [35].	There is a relatively poor understanding of factors influencing the encapsulation process and there are limitations on the type of organic phases and surfactants used [46]. In addition, particles present low thermal stability [49].	55–1300 nm	Citrate buffer and medium-chain triglyceride	Carvacrol	[46,50]
				112–594 nm	Polyoxyethylene (20, 40, 60, and 80) and castor oil ether	D-limonene	[51]
	Miniemulsion Polymerization	Fine monomer droplets are produced by the action of high shear (ultrasonic waves or high-speed homogenizer), so polymer particles are obtained via oil-soluble initiators, through droplet nucleation. However, some monomers have slight solubility in water and a co-stabilizer be also used, in addition to surfactants, to avoid coalescence (Ostwald ripening) [35].	It is a technique still under improvement without complete knowledge of its mechanism. Additionally, it can present a wide particle size distribution [35].	53.25–247.6 nm	Polysorbate 80 (Tween^®^ 80)	*Melaleuca alternifolia* (tea tree oil), *Vitis vinifera* (grapes seeds oil), and *Punica granatum* (pomegranate fruit peel oil)	[52]
Chemical Processes	Melt -Dispersion	The active component is melted in water at a temperature above the melting point and emulsified in a high-pressure homogenizer [53].	Depending on the temperature set, it can volatilize core components [54].	240 nm	Poly-ethylene glycol (PEG)	Garlic essential oil	[54]
	In situ polymerization	Direct polymerization of a solution of monomers or oligomers is carried out on the core material surface. Deposition and precipitation are controlled by precipitants or changes in pH and temperature [7,55].	There is little knowledge on precise control of the microencapsulation process, affecting the rational design and efficiency of the microcapsules [55]	20–110 µm	Phenol and formaldehyde	Neem oil	[56]
				30–600 µm	Phenol, formalin, poly(vinyl alcohol) (PVA), butanol and sodium lauryl sulfate, cardanol, xylene, and resorcinol.	Karanja oil	[57]
	Complex coacervation	This technique relies upon a decrease in solubility of the coating polymer when a third component is added to the polymer solution. Two oppositely charged polymers form a wall around the active ingredient, due to the complexation of oppositely charged polyelectrolytes [44,58,59,60].	This technique usually requires toxic reagents for the coacervate shell [60].	35–50 µm	Gelatin and gum Arabic	*Metarhizium anisopliae*	[61]
Physical Processes	Spray Drying	The core material is homogenized with the carrier, usually an aqueous solution, and then set in a spray dryer Tiny droplets are formed and, by contact with the hot gas, water is evaporated, obtaining a powder or granular product [42,62].	Highly cost equipment and expensive powder recovery process. High heat consumption and low thermal efficiency [63].	1.10–2.09 µm	Chitosan and sodium lignosulfonate	Spinosad	[64]
				28.84–52.88 µm	PVA, gum Arabic, and whey protein isolate/maltodextrin	Neem seed oil	[65]
				15–20 µm	Maltodextrin, gum Arabic	*Trichoderma harzianum*	[66]
Physical Processes	Fluidized Bed Coating (FBC)	In this method, particles with different diameters are moved around in a fluidized bed and sprayed with a liquid. The solution, either aqueous or organic, evaporates and forms a coating layer around the active ingredient [67].	FBC can be applied to a limited range of active ingredients since it degrades temperature-sensitive active compounds [49]. It presents difficulties in processing needle or platelet-shaped particles [68].	-	Biomass	*Metarhizium brunneum, Cordyceps fumosorosea* and *Beauveria bassiana*	[69]
	Ionic Gelation	This technique is based on ionic interactions between charged groups of the polymer and charged groups of the crosslinking agent [70,71].	It can result in nanoparticles and microparticles with a fragile particulate system, high dispersibility index, and few sites to modify the surface for functional moieties attachment [71].	-	Alginate, CaCl_2,_ and glycerol	*H. bacteriophora*	[72]
				-	Alginate—multivalent counterions (calcium chloride, zinc sulfate, copper sulfate, cobalt chloride, and ferric chloride)	*Bacillus thuringiensis* var. *israelensis*	[73]

**Table 3:** Biopesticide encapsulation using supercritical CO_2_.

Supercritical Technology	Active Ingredient	scCO_2_ role	Material	Solvent	Temperature (°C)	Pressure (MPa)	Particle Size	Reference
RESS	*Atractylodes macrocephala* essential oil	Solvent	Phosphatidylcholine and cholesterol	Ethanol	65	30	173 nm	[83,84]
PGSS	*Cydia pomonella* granulovirus (CpGV)	Solute	Palm oil-based fat, lecithin-based surfactant, and modified TiO_2_ as a UV protectant.	-	65	10	<85 µm	[85]
	Lavandin oil	Solute	OSA starch and PEG	-	64–74	7.6–8.4	21–49 µm	[86]
SSI	1-octen-3-ol	Solvent	Low-Density Polyethylene (LPDE) films	-	45	7.5–14.5	-	[87]
	Thymoquinone and R-(+)-pulegone	Solvent	LDPE films	-	45	10–15	-	[88]
	Pyrethrins	Solvent	Polypropylene films	-	35–55	10–40	-	[89]
SAA	Rotenone	Cosolute	PEG, alginate, and Polyvinylpyrrolidone (PVP)	Acetone, water, and ethyl acetate	40–85	8–11	0.6–1.5 µm	[90]
SAF	Ryanodol	Antisolvent	-	Ethanol	35	15	5 µm	[91]
Supercritical Phase Inversion	Quercetin	Solvent	Cellulose acetate	Acetone	45–50	10–20	-	[92]

## References

[1] Styring A.K., Charles M., Fantone F., Hald M.M., McMahon A., Meadow R.H., Nicholls G.K., Patel A.K., Pitre M.C., Smith A. (2017). Isotope evidence for agricultural extensification reveals how the world’s first cities were fed. Nat. Plants.

[2] Samada L.H., Tambunan U.S.F. (2020). Biopesticides as promising alternatives to chemical pesticides: A review of their current and future status. Online J. Biol. Sci..

[3] Singh A., Dhiman N., Kar A.K., Singh D., Purohit M.P., Ghosh D., Patnaik S. (2020). Advances in controlled release pesticide formulations: Prospects to safer integrated pest management and sustainable agriculture. J. Hazard. Mater..

[4] Kumar S., Singh A. (2015). Biopesticides: Present status and the future prospects. J. Biofertil. Biopestic..

[5] Freimoser F.M., Rueda-Mejia M.P., Tilocca B., Migheli Q. (2019). Biocontrol yeasts: Mechanisms and applications. World J. Microbiol. Biotechnol..

[6] De Silva N.I., Brooks S., Lumyong S., Hyde K.D. (2019). Use of endophytes as biocontrol agents. Fungal Biol. Rev..

[7] Roy A., Singh S.K., Bajpai J., Bajpai A.K. (2014). Controlled pesticide release from biodegradable polymers. Cent. Eur. J. Chem..

[8] Maes C., Bouquillon S., Fauconnier M.-L. (2019). Encapsulation of essential oils for the development of biosourced pesticides with controlled release: A review. Molecules.

[9] Rad H.B., Sabet J.K., Varaminian F. (2019). Study of solubility in supercritical fluids: Thermodynamic concepts and measurement methods—A review. Braz. J. Chem. Eng..

[10] Montes A., Gordillo M.D., Pereyra C., de la Ossa E.J.M. (2011). Particles formation using supercritical fluids. Mass Transfer—Advanced Aspects.

[11] Keven Silva E., Angela A., Meireles M. (2014). Encapsulation of food compounds using supercritical technologies: Applications of supercritical carbon dioxide as an antisolvent. Food Public Health.

[12] Abbey L., Abbey J., Leke-aladekoba A., Iheshiulo E.M., Ijenyo M. (2019). Biopesticides and Biofertilizers: Types, Production, Benefits, and Utilization. Byproducts from Agriculture and Fisheries: Adding Value for Food, Feed, Pharma, and Fuels.

[13] Senthil-Nathan S. (2015). A review of biopesticides and their mode of action against insect pests. Environmental Sustainability: Role of Green Technologies.

[14] Liu X., Cao A., Yan D., Ouyang C., Wang Q. (2019). Overview of mechanisms and uses of biopesticides. Int. J. Pest Manag..

[15] Damalas C.A., Koutroubas S.D. (2018). Current status and recent developments in biopesticide use. Agriculture.

[16] Allan G.G., Miller T.A. (1987). Long-Acting Pyrethrum/Pyrethroid Based Pesticides with Silicone Stabilizers. U.S. Patent.

[17] Markus A., Schuster D., Linder C., Strongin P. (2015). US9101143B2—Formulations Containing Microencapsulated Essential Oils. U.S. Patent.

[18] Miresmailli S., Ojha H.D., Drury J.W. (2018). US10058092B2—Apparatus and Method for Controlled Release of Botanical Fumigant Pesticides. U.S. Patent.

[19] Wright J.E., Chandler L.D. (1995). US005413784A—Biopesticide Composition and Process for Controlling Insect Pests. U.S. Patent.

[20] Valent BioSciences XenTari®. https://www.valentbiosciences.com/cropprotection/products/xentari/.

[21] Valent BioSciences DiPel®. https://www.valentbiosciences.com/cropprotection/products/dipel/.

[22] Xi’an Neo Biotech Co L. Bacillus Thuringiensis. http://www.neobio.site/chemicalfactory/Bacillus_Thuringiensis-44.

[23] Arbico Organics Milky Spore Powder—Safety Data Sheet. https://www.arbico-organics.com/product/milky-spore-powder-bacillus-popilliae-control-japanese-beetle/organic-lawn-care.

[24] Marrone Bio Innovations Grandevo®. https://marronebio.com/products/grandevo/.

[25] Bayer Serenade®. https://www.agro.bayer.com.br/home/fungicida-serenade.

[26] Valent Actinovate® AG. https://www.valent.com/products/actinovate-ag.

[27] Bonide Neem Oil. https://www.arbico-organics.com/product/neem-oil-concentrate-insecticide-fungicide-miticide/neem-oil-insecticides.

[28] Stocken Timorex Gold. https://www.syngenta.com.au/product/crop-protection/timorex-gold.

[29] Monterey Take Down Garden Spray. https://www.montereylawngarden.com/product/take-down-garden-spray/.

[30] Brandt Kaligreen. https://brandt.co/lines/kaligreen/.

[31] Marrone Bio Innovations Regalia. https://marronebio.com/products/regalia/.

[32] Isman M.B. (2016). Pesticides based on plant essential oils: Phytochemical and practical considerations. ACS Symp. Ser..

[33] Liu B., Wang Y., Yang F., Wang X., Shen H., Cui H., Wu D. (2016). Construction of a controlled-release delivery system for pesticides using biodegradable PLA-based microcapsules. Colloids Surf. B Biointerfaces.

[34] Tsuda N., Ohtsubo T., Fuji M. (2012). Preparation of self-bursting microcapsules by interfacial polymerization. Adv. Powder Technol..

[35] Gharieh A., Khoee S., Reza A. (2019). Emulsion and miniemulsion techniques in preparation of polymer nanoparticles with versatile characteristics. Adv. Colloid Interface Sci..

[36] Couto R., Alvarez V., Temelli F. (2017). Encapsulation of Vitamin B2 in solid lipid nanoparticles using supercritical CO2. J. Supercrit. Fluids.

[37] Sonawane S.H., Bhanvase B.A., Sivakumar M., Potdar S.B. (2020). Current Overview of Encapsulation. Encapsulation of Active Molecules and Their Delivery System.

[38] McClements D.J. (2018). Recent developments in encapsulation and release of functional food ingredients: Delivery by design. Curr. Opin. Food Sci..

[39] Bao C., Jiang P., Chai J., Jiang Y., Li D., Bao W. (2019). The delivery of sensitive food bioactive ingredients: Absorption mechanisms, influencing factors, encapsulation techniques and evaluation models. Food Res. Int..

[40] Carneiro H.C.F., Tonon R.V., Grosso C.R.F., Hubinger M.D. (2013). Encapsulation efficiency and oxidative stability of flaxseed oil microencapsulated by spray drying using different combinations of wall materials. J. Food Eng..

[41] Slattery M., Harper B., Harper S. (2019). Pesticide encapsulation at the nanoscale drives changes to the hydrophobic partitioning and toxicity of an active ingredient. Nanomaterials.

[42] Cocero M.J., Martín Á., Mattea F., Varona S. (2009). Encapsulation and co-precipitation processes with supercritical fluids: Fundamentals and applications. J. Supercrit. Fluids J..

[43] Li M., Rouaud O., Poncelet D. (2008). Microencapsulation by solvent evaporation: State of the art for process engineering approaches. Int. J. Pharm. J..

[44] Huang B., Chen F., Shen Y., Qian K., Wang Y. (2018). Advances in targeted pesticides with environmentally responsive controlled release by nanotechnology. Nanomaterials.

[45] Klettenhammer S., Ferrentino G., Morozova K., Scampicchio M. (2020). Novel technologies based on supercritical fluids for the encapsulation of food grade bioactive compounds. Foods.

[46] Chang Y., Mclandsborough L., McClements D.J. (2013). Physicochemical properties and antimicrobial efficacy of carvacrol nanoemulsions formed by spontaneous emulsification. J. Agric. Food Chem..

[47] El-Hoshoudy A.N.M.B. (2018). Emulsion Polymerization Mechanism. Recent Research in Polymerization.

[48] José M.A. (2004). Emulsion polymerization: From fundamental mechanisms to process developments. J. Polym. Sci. Part A Polym. Chem..

[49] Raza Z.A., Khalil S., Ayub A., Banat I.M. (2020). Recent developments in chitosan encapsulation of various active ingredients for multifunctional applications. Carbohydr. Res..

[50] Campos E.V.R., Proença P.L.F., Oliveira J.L., Pereira A.E.S., De Morais Ribeiro L.N., Fernandes F.O., Gonçalves K.C., Polanczyk R.A., Pasquoto-Stigliani T., Lima R. (2018). Carvacrol and linalool co-loaded in β-cyclodextrin-grafted chitosan nanoparticles as sustainable biopesticide aiming pest control. Sci. Rep..

[51] Feng J., Wang R., Chen Z., Zhang S., Yuan S., Cao H., Jafari S.M., Yang W. (2020). Formulation optimization of D-limonene-loaded nanoemulsions as a natural and efficient biopesticide. Colloids Surf. A Physicochem. Eng. Asp..

[52] Wahba T.F. (2020). Antifeedant activity of three essential oils and their nanoemulsions against antifeedant activity of three essential oils and their nanoemulsions against the rice weevil *Sitophilus oryzae* (L.). Egypt. Sci. J. Pestic..

[53] Pan Z., Cui B., Zeng Z., Feng L., Liu G., Cui H., Pan H. (2015). Lambda-cyhalothrin nanosuspension prepared by the melt emulsification-high pressure homogenization method. J. Nanomater..

[54] Yang F.L., Li X.G., Zhu F., Lei C.L. (2009). Structural characterization of nanoparticles loaded with garlic essential oil and their insecticidal activity against Tribolium castaneum (Herbst) (Coleoptera: Tenebrionidae). J. Agric. Food Chem..

[55] Nguon O., Lagugné-labarthet F., Brandys F.A., Li J., Gillies E.R. (2018). Microencapsulation by in situ polymerization of amino resins. Polym. Rev..

[56] Bagle A.V., Jadhav R.S., Gite V.V., Hundiwale D.G., Mahulikar P.P. (2013). Controlled release study of phenol formaldehyde microcapsules containing neem oil as an insecticide. Int. J. Polym. Mater. Polym. Biomater..

[57] Hedaoo R.K., Gite V.V. (2014). Renewable resource-based polymeric microencapsulation of natural pesticide and its release study: An alternative green approach. RSC Adv..

[58] Wang B., Akanbi T.O., Agyei D., Holland B.J., Barrow C.J. (2018). Encapsulation and delivery tool for hydrophobic biofunctional compounds. Role of Materials Science in Food Bioengineering.

[59] Eghbal N., Choudhary R. (2018). Complex coacervation: Encapsulation and controlled release of active agents in food systems. LWT Food Sci. Technol..

[60] Petrusic S., Koncar V. (2016). Controlled Release of Active Agents from Microcapsules Embedded in Textile Structures.

[61] Qiu H.L., Fox E.G.P., Qin C.S., Zhao D.Y., Yang H., Xu J.Z. (2019). Microcapsuled entomopathogenic fungus against fire ants, Solenopsis invicta. Biol. Control.

[62] Chandralekha A., Tavanandi A.H., Amrutha N., Hebbar H.U. (2016). Encapsulation of yeast (Saccharomyces cerevisiae) by spray drying for extension of shelf life. Dry. Technol..

[63] Sosnik A., Seremeta K.P. (2015). Advantages and challenges of the spray-drying technology for the production of pure drug particles and drug-loaded polymeric carriers. Adv. Colloid Interface Sci..

[64] Pérez-Landa I.D., Bonilla-Landa I., Monribot-Villanueva J.L., Ramírez-Vázquez M., Lasa R., Ramos-Torres W., Olivares-Romero J.L., Barrera-Méndez F. (2021). Photoprotection and release study of spinosad biopolymeric microparticles obtained by spray drying. Powder Technol..

[65] Sittipummongkol K., Chuysinuan P., Techasakul S., Pisitsak P., Pechyen C. (2018). Core shell microcapsules of neem seed oil extract containing azadirachtin and biodegradable polymers and their release characteristics. Polym. Bull..

[66] Muñoz-Celaya A.L., Ortiz-García M., Vernon-Carter E.J., Jauregui-Rincón J., Galindo E., Serrano-Carreón L. (2012). Spray-drying microencapsulation of Trichoderma harzianum conidias in carbohydrate polymers matrices. Carbohydr. Polym..

[67] Dewettinck K., Huyghebaert A. (1999). Fluidized bed coating in food technology. Trends Food Sci. Technol..

[68] Chua K.J., Chour S.K., Sun D.-W. (2005). New hybrid drying technologies. Emerging Technologies for Food Processing.

[69] Stephan D., Bernhardt T., Buranjadze M., Seib C., Schäfer J., Maguire N., Pelz J. (2020). Development of a fluid-bed coating process for soil-granule-based formulations of Metarhizium brunneum, Cordyceps fumosorosea or Beauveria bassiana. J. Appl. Microbiol..

[70] Fan W., Yan W., Xu Z., Ni H. (2012). Formation mechanism of monodisperse, low molecular weight chitosan nanoparticles by ionic gelation technique. Colloids Surf. B Biointerfaces.

[71] Kunjachan S., Jose S. (2010). Understanding the mechanism of ionic gelation for the synthesis of chitosan nanoparticles using qualitative techniques. Asian J. Pharm..

[72] Jaffuel G., Sbaiti I., Turlings T.C.J. (2020). Encapsulated entomopathogenic nematodes can protect maize plants from diabrotica balteata larvae. Insects.

[73] Prabakaran G., Hoti S.L. (2008). Immobilization of alginate-encapsulated Bacillus thuringiensis var. israelensis containing different multivalent counterions for mosquito control. Curr. Microbiol..

[74] Bertucco A., Vetter G., Sie S.T. (2001). High Pressure Process Technology: Fundamentals and Applications.

[75] Yousefi M., Rahimi-Nasrabadi M., Mirsadeghi S., Pourmortazavi S.M. (2020). Supercritical fluid extraction of pesticides and insecticides from food samples and plant materials. Crit. Rev. Anal. Chem..

[76] Martín L., Marqués J.L., González-Coloma A., Mainar A.M., Palavra A.M.F., Urieta J.S. (2012). Supercritical methodologies applied to the production of biopesticides: A review. Phytochem. Rev..

[77] Chakravarty P., Famili A., Nagapudi K., Al-Sayah M.A. (2019). Using Supercritical Fluid Technology as a Green Alternative during the Preparation of Drug Delivery Systems. Pharmaceutics.

[78] Tabernero A., Martín del Valle E.M., Galán M.A. (2012). Supercritical fluids for pharmaceutical particle engineering: Methods, basic fundamentals and modelling. Chem. Eng. Process. Process Intensif..

[79] Kanakubo M., Aizawa T., Kawakami T., Sato O., Ikushima Y., Hatakeda K., Saito N. (2000). Studies on solute-solvent interactions in gaseous and supercritical carbon dioxide by high-pressure H NMR spectroscopy. J. Phys. Chem. B.

[80] Santos D.T., Meireles M.A.A. (2013). Micronization and encapsulation of functional pigments using supercritical carbon dioxide. J. Food Process Eng..

[81] Reverchon E. (2002). Supercritical-assisted atomization to produce micro-and/or nanoparticles of controlled size and distribution. Ind. Eng. Chem. Res..

[82] Prosapio V., De Marco I., Reverchon E. (2018). Supercritical antisolvent coprecipitation mechanisms. J. Supercrit. Fluids.

[83] Wen Z., Liu B., Zheng Z., You X., Pu Y., Li Q. (2010). Preparation of liposomes entrapping essential oil from Atractylodes macrocephala Koidz by modified RESS technique. Chem. Eng. Res. Des..

[84] Chu S.S., Jiang G.H., Liu Z.L. (2011). Insecticidal compounds from the essential oil of Chinese medicinal herb Atractylodes chinensis. Pest Manag. Sci..

[85] Pemsel M., Schwab S., Scheurer A., Freitag D., Schatz R., Schlücker E. (2010). Advanced PGSS process for the encapsulation of the biopesticide Cydia pomonella granulovirus. J. Supercrit. Fluids.

[86] Varona S., Kareth S., Cocero M.J. Encapsulation of essentials oils using biopolymers for their use in ecological agriculture. Proceedings of the 9th International Symposium on Supercritical Fluids.

[87] Herrera J.M., Gañán N.A., Goñi M.L., Zygadlo J.A., Martini R.E. (2018). Active LDPE films loaded with biopesticides by supercritical CO_2_-assisted impregnation for stored grain protection. Food Packag. Shelf Life.

[88] Goñi M.L., Gañán N.A., Herrera J.M., Strumia M.C., Andreatta A.E., Martini R.E. (2017). Supercritical CO2 impregnation of LDPE films with terpene ketones as biopesticides against corn weevil (*Sitophilus zeamais*). J. Supercrit. Fluids.

[89] Maya C., Fernández-Ponce M.T., Casas L., Mantell C., Martínez de la Ossa E.J. (2021). A comparative analysis on the impregnation efficiency of a natural insecticide into polypropylene films by means of batch against semi-continuous techniques using CO_2_ as solvent. J. Supercrit. Fluids.

[90] Martin L., Liparoti S., Della Porta G., Adami R., Marqués J.L., Urieta J.S., Mainar A.M., Reverchon E. (2013). Rotenone coprecipitation with biodegradable polymers by supercritical assisted atomization. J. Supercrit. Fluids.

[91] Martín L., González-Coloma A., Adami R., Scognamiglio M., Reverchon E., Della Porta G., Urieta J.S., Mainar A.M. (2011). Supercritical antisolvent fractionation of ryanodol from Persea indica. J. Supercrit. Fluids.

[92] Baldino L., González-Garcinuño Á., Tabernero A., Cardea S., Del Valle E.M.M., Reverchon E. (2021). Production of fungistatic porous structures of cellulose acetate loaded with quercetin, using supercritical CO_2_. J. Supercrit. Fluids.

[93] Reverchon E., Adami R. (2006). Nanomaterials and supercritical fluids. J. Supercrit. Fluids.

[94] Montes A., Merino R., De Los Santos D.M., Pereyra C., Martínez De La Ossa E.J. (2017). Micronization of vanillin by rapid expansion of supercritical solutions process. J. CO_2_ Util..

[95] Tokunaga S., Ono K., Ito S., Sharmin T., Kato T., Irie K., Mishima K., Satho T., Harada T. (2021). Microencapsulation of drug with enteric polymer Eudragit L100 for controlled release using the particles from gas saturated solutions (PGSS) process. J. Supercrit. Fluids.

[96] Goñi M.L., Gañán N.A., Strumia M.C., Martini R.E. (2016). Eugenol-loaded LLDPE films with antioxidant activity by supercritical carbon dioxide impregnation. J. Supercrit. Fluids.

[97] Wu H., Chen H., Lee H. (2019). Controlled release of theophylline-chitosan composite particles prepared using supercritical assisted atomization. Braz. J. Chem. Eng..

[98] Santo I.E., Campardelli R., Albuquerque E.C., de Melo S.V., Della Porta G., Reverchon E. (2014). Liposomes preparation using a supercritical fluid assisted continuous process. Chem. Eng. J..

[99] Adami R., Liparoti S., Reverchon E. (2011). A new supercritical assisted atomization configuration, for the micronization of thermolabile compounds. Chem. Eng. J..

[100] Baldino L., Cardea S., Reverchon E. (2020). Supercritical phase inversion: A powerful tool for generating cellulose acetate-AgNO3 antimicrobial membranes. Materials.

[101] Baldino L., Cardea S., Reverchon E. (2015). Antimicrobial membranes produced by supercritical assisted phase inversion. Chem. Eng. Trans..

[102] Tabernero A., Cardea S. (2020). Supercritical carbon dioxide techniques for processing microbial exopolysaccharides used in biomedical applications. Mater. Sci. Eng. C.

[103] Ulker Z., Erkey C. (2014). An emerging platform for drug delivery: Aerogel based systems. J. Control. Release.

[104] Gurikov P., Raman S.P., Griffin J.S., Steiner S.A., Smirnova I. (2019). 110th Anniversary: Solvent exchange in the processing of biopolymer aerogels: Current status and open questions. Ind. Eng. Chem. Res..

[105] García-González C.A., Sosnik A., Kalmar J., Marco I., Erkey C., Conceiro A., Alvarez-Lorenzo C. (2021). Aerogels in drug delivery: From design to application. J. Control. Release.

[106] Tkalec G., Pantić M., Novak Z., Knez Ž. (2015). Supercritical impregnation of drugs and supercritical fluid deposition of metals into aerogels. J. Mater. Sci..

[107] Schwertfeger F., Zimmermann A., Frisch G. (2010). US7674476B1—Use of Aerogels in Agriculture. U.S. Patent.

[108] Reverchon E., De Marco I., Torino E. (2007). Nanoparticles production by supercritical antisolvent precipitation: A general interpretation. J. Supercrit. Fluids.

[109] Reverchon E., De Marco I. (2011). Mechanisms controlling supercritical antisolvent precipitate morphology. Chem. Eng. J..

[110] Franco P., De Marco I. (2020). Supercritical antisolvent process for pharmaceutical applications: A review. Processes.

[111] Oliveira D.A., Mezzomo N., Gomes C., Ferreira S.R.S. (2017). Encapsulation of passion fruit seed oil by means of the supercritical antisolvent process. J. Supercrit. Fluids.

[112] Flaherty R.J., Nshime B., DeLaMarre M., DeJong S., Scott P., Lantz A.W. (2013). Cyclodextrins as complexation and extraction agents for pesticides from contaminated soil. Chemosphere.

[113] Tabernero A., Martín Del Valle E.M., Galán M.A. (2013). Experimental and theoretical analysis of the operating parameters for precipitating acetaminophen and tretinoin with solution enhanced dispersion by supercritical fluids. Ind. Eng. Chem. Res..

[114] Reverchon E., Adami R., Caputo G. (2006). Supercritical assisted atomization: Performance comparison between laboratory and pilot scale. J. Supercrit. Fluids.

[115] York P., Kompella U.B., Shekunov B.Y. (2004). Supercritical Fluid Technology for Drug Product Development.

[116] Kankala R.K., Zhang Y.S., Wang S.B., Lee C.H., Chen A.Z. (2017). Supercritical fluid technology: An emphasis on drug delivery and related biomedical applications. Adv. Healthc. Mater..

